# Traditional risk factors and cancer-related factors associated with cardiovascular disease risk in head and neck cancer patients

**DOI:** 10.3389/fcvm.2022.1024846

**Published:** 2023-01-12

**Authors:** Amrita Mukherjee, Howard W. Wiener, Russell L. Griffin, Carrie Lenneman, Arka Chatterjee, Lisle M. Nabell, Cora E. Lewis, Sadeep Shrestha

**Affiliations:** ^1^Department of Epidemiology, School of Public Health, University of Alabama at Birmingham School of Public Health, Birmingham, AL, United States; ^2^Department of Research & Evaluation, Kaiser Permanente Southern California, Pasadena, CA, United States; ^3^Division of Cardiovascular Disease, University of Alabama at Birmingham School of Medicine, Birmingham, AL, United States; ^4^Department of Medicine, Sarver Heart Center, University of Arizona Health Sciences, Tucson, AZ, United States; ^5^Division of Hematology and Oncology, University of Alabama at Birmingham School of Medicine, Birmingham, AL, United States

**Keywords:** head and neck cancer, cardiovascular disease, traditional risk factors, hypertension, electronic health records

## Abstract

**Background:**

Risk of incident cardiovascular disease (CVD) in head and neck squamous cell carcinoma (HNSCC) patients is under-reported. We assessed the association of HNSCC-related factors and traditional risk factors with 1- and 5-year CVD risk in HNSCC patients without prevalent CVD at cancer diagnosis.

**Methods:**

A clinical cohort of 1,829 HNSCC patients diagnosed between 2012 and 2018, at a National Cancer Institute (NCI)-designated cancer center was included. Information on HNSCC-related factors [HNSCC anatomical subsite, stage at diagnosis, treatment, and tumor human papillomavirus (HPV) status] were extracted from the tumor registry. Data on traditional risk factors (hypertension, dyslipidemia, diabetes, tobacco smoking status, and obesity) were extracted from the electronic health records system (EHR) at baseline (HNSCC diagnosis). A composite of ischemic heart disease, heart failure, and ischemic stroke was the outcome of interest in time to event analysis. Hazard ratio (HR) (95% CI) were reported with death as a competing risk.

**Results:**

In patients diagnosed with HNSCC, 10.61% developed incident CVD events by 1-year post cancer diagnosis. One-year CVD risk was lower in patients using antihypertensive medications at baseline, compared to patients without baseline hypertension [HR (95% CI): 0.41 (0.24–0.61)]. One-year CVD risk was high in patients receiving HNSCC surgery. Patients receiving radiation therapy had a higher 5-year CVD risk than surgery patients [HR (95% CI): 2.17 (1.31–3.04)]. Patients using antihypertensive medications had a lower 5-year CVD risk than patients without baseline hypertension [HR (95% CI): 0.45 (0.22–0.75)]. Older age and diabetes were associated with increased 1- and 5-year CVD risk. HPV-negative patients were older (*p* 0.006) and had a higher 5-year cumulative incidence of CVD (*p* 0.013) than HPV-positive patients.

**Conclusion:**

Traditional risk factors and cancer-related factors are associated with CVD risk in HNSCC patients. Future research should investigate the role of antihypertensive medications in reducing CVD risk in HNSCC patients.

## Introduction

Head and neck cancer (HNC) accounts for approximately 14,600 deaths in the United States (US) annually (1). With advances in cancer screening and use of multimodality treatment, survival has improved in HNC patients (2). However, with increased survival, the disease burden of comorbidities and cancer treatment related side-effects have also increased. Cancer patients and survivors have a higher burden of cardiovascular disease (CVD) than the age-adjusted general population (3–5). Cardiotoxic effects of cancer therapies, especially the usage of anthracyclines, platinum-based agents, targeted kinase inhibitors and 5-fluorouracil in aggravating left ventricular (LV) dysfunction, heart failure, stroke, or myocardial infarction have been reported in cancer patients, including patients with HNC (6–9). Increased risk of cerebrovascular events in HNC patients receiving radiation therapy is also established (10–12). Inflammation, vascular damage, and accelerated atherosclerosis following cancer radiation are responsible for increased risk of stroke or transient ischemic attacks (13). Peri- and post-operative CVD complications in HNC surgery are not uncommon (14–17).

Cancer and CVD share common risk factors including obesity, smoking, and diabetes. Older age and inflammation also play important roles in the pathophysiology of CVD in cancer patients (18). Modifiable risk factors like hypertension, dyslipidemia, and obesity are associated with increased CVD risk in adult survivors of childhood cancer (19). While several studies have reported on CVD morbidity and mortality in cancer patients (19–22), association of traditional risk factors with CVD risk vary by cancer type (22). Literature assessing CVD risk in HNC patients is limited (10) and studies are mostly restricted to older HNC patients who had prevalent CVD events (23, 24).

Our objective was to assess association of head and neck squamous cell carcinoma (HNSCC)-related factors and traditional risk factors with incident CVD events (ischemic heart disease, heart failure, and ischemic stroke) in patients without a prior history of CVD at HNSCC diagnosis.

## Materials and methods

### Study population

**FIGURE 1:**
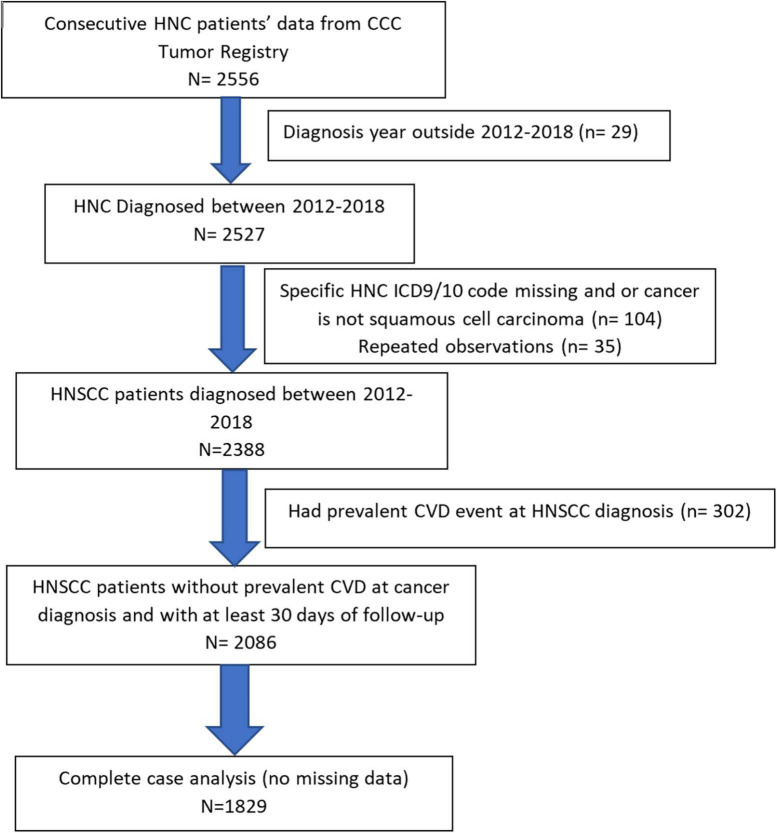
Flowchart of head and neck squamous cell carcinoma (HNSCC) study population based on inclusion/exclusion criteria is presented.

In this clinical cohort, 1,829 consecutive HNSCC patients diagnosed between January 2012 and December 2018, at the University of Alabama at Birmingham (UAB) hospital system and O’Neal comprehensive cancer center (CCC) were included (Figure 1). HNSCC patients were identified from the UAB CCC tumor registry and electronic health records (EHR) system using ICD 9/10 diagnosis codes. HNSCC diagnosis codes and histology were confirmed by physician notes and ICD-O-3 histology codes. Patients were included if they met the following inclusion criteria:

a) Confirmed ICD9/10 codes for HNC diagnosis [ICD9 codes: 140.–149., 160. (except 160.1), 161.; ICD10 codes: (C00–C14, C30.0, C31, and C32.)] and histologically confirmed squamous cell carcinoma of head and neck (ICD-O-3 histology codes: 805–808).
b) Had HNSCC diagnosis date.
c) 18 years or above at HNSCC diagnosis (baseline).
d) Did not have any prevalent CVD (ischemic heart disease, heart failure, or ischemic stroke) at baseline.
e) Did not have missing data.

Follow-up data from the EHR were extracted until 31st December, 2020 (end of study period). De-identified data were analyzed. The study was approved by the UAB Institutional Review Board (IRB) and CCC, and a waiver of written informed consent was granted. The first author (AM) had full access to all the data in the study and takes responsibility for its integrity and data analyses.

### Outcome of interest

Our outcome of interest was a composite of incident ischemic heart disease, heart failure, and ischemic stroke, whichever occurred first. CVD outcomes were identified and recorded from the EHR at each clinic visit using the following ICD 9/10 codes:

a) ICD-9 codes: 410.–414. (ischemic heart disease), 428. (heart failure), 433.–434. (ischemic stroke if cerebral infarction present).
b) ICD-10 codes: I20.–I25. (ischemic heart disease), I50. (heart failure), and I63.–I64. (ischemic stroke if cerebral infarction present).

For a sub-sample of the study population (*n* = 200), CVD outcomes were validated by reviewing medical charts (kappa 0.78).

### HNSCC-related factors

Data on HNSCC anatomical subsite, stage at diagnosis, treatment, and tumor human papillomavirus (HPV) status were extracted. HNSCC anatomical subsite was categorized as: oral cavity, oropharynx, hypopharynx, nasopharynx-nasal cavity, larynx, and major salivary. HNSCC clinical stage at diagnosis was categorized as: early (stages 0/I/II)/advanced (stages III/IV)/other, based on the American Joint Committee on Cancer TNM classification, 7th edition (25). Patients with “incomplete/unstageable/not defined” TNM classification records were grouped in the “other” category. HNSCC treatment had the following categories: only surgery, only chemotherapy, only radiation therapy, chemoradiation, surgery with chemo/radiation, and no HNSCC treatment. Patients in the “no HNSCC treatment” category did not receive surgery, chemotherapy, or radiation for HNSCC, but might have received palliative care or alternate therapy. Data on type of surgery (local excision/wide excision/radical surgery) were also extracted. Information on tumor HPV status was available for a subsample of the study population (*n* = 567) in the tumor registry. Patients were categorized as HPV positive/negative, based on presence of high-risk HPV types.

### Traditional risk factors

Baseline tobacco and alcohol use were categorized into three groups based on patients’ self-reports- current users, former users and never. Body mass index (BMI) categories included- non-obese (BMI < 30.0 kg/m^2^) and obese (BMI ≥ 30.0 kg/m^2^). For clinically diagnosed CVD risk factors- hypertension, dyslipidemia, and diabetes mellitus, a combination of ICD 9/10 codes and medication use/pharmacy records was used (Supplementary Table 1). Baseline CVD clinical risk factors were included as categorical variables and had the following categories- absent, present with medication use, and present without medication use.

### Other covariates

Information on self-reported socio-demographic variables- age, sex, race, marital status, and geographic location/residence were extracted from the EHR at baseline. Age was included as a continuous variable, as well as a categorical variable- ≤ 45 years, 46 to < 65 years, and 65 years or above. Geographic residence was categorized as rural or urban, based on patients’ residential zip-codes. Rural and urban counties were defined based on 2010 United States Census Bureau’s urban-rural classification (26).

### Statistical analysis

Distribution of baseline demographic characteristics, traditional CVD risk factors, and HNSCC-related variables were reported using median (IQR) or frequency (percentage), as appropriate. For time to event analysis, follow-up started after 30 days post HNSCC diagnosis to make sure that prevalent CVD cases were not included. Patients were followed until they developed the first CVD event (a composite of incident ischemic heart disease, heart failure, and ischemic stroke, whichever occurred first), death, loss to follow-up, or end of study, whichever occurred first.

Proportional hazard assumptions were checked using variable*time interactions and Kolmogorov–Supremum test. Parametric accelerated failure time (AFT) survival models with Weibull distribution were used to assess 1- and 5-year risk of incident CVD events, as the proportional hazards assumptions were not met. Death was treated as a competing risk and the AFT models were censored for death, loss to follow-up, or end of study (follow-up), whichever occurred first. Hazard ratio (HR) was calculated from AFT-Weibull models by exponentiating −αβ, where α is Weibull shape and β is the parameter estimate (HR = *e*^–^^α^ ^β^). In the adjusted models, variables with clinical/biological relevance and variables with unadjusted *p*-value ≤ 0.10 were included. Cumulative incidence of CVD events was plotted.

In sensitivity analysis, separate AFT models were analyzed for association of traditional CVD risk factors and HNSCC-related factors with incident CVD in patients aged < 65 years and in patients ≥ 65 years. Cutoff for age was set at 65 years, as the average age for first CVD event in US men is ∼65 years (27). Association of HPV status with CVD, along with CVD cumulative incidence plots by HPV status were reported. Level of statistical significance was set at 0.05. Hazard ratio (HR, 95% CI) and two-sided *p*-values were reported. All statistical analyses were performed in SAS 9.4 (Cary, NC, USA).

## Results

Eighteen hundred twenty-nine HNSCC patients were included in the study. By 1-year post HNSCC diagnosis, 10.61% of all HNSCC patients developed incident CVD events. In patients with incident CVD by 1-year, 83.51% patients had ischemic heart disease, 11.34% had heart failure and 5.15% patients had ischemic stroke as their first CVD event. Baseline demographic characteristics, traditional risk factors, and HNSCC-related factors of all patients and patients by incident CVD status at 1-year post HNSCC diagnosis are reported in Table 1. Patients who developed CVD by 1-year were older than patients who did not [median age 67.0 (61.0–74.0) vs. 60.0 (53.0–67.0) years, *p* < 0.001]. Hypertension was the most common CVD clinical risk factor at baseline (54.02%), followed by dyslipidemia (26.74%) and diabetes (13.04%). Nearly half of the HNSCC patients (49.54%) were diagnosed at an advanced AJCC clinical stage. Surgery was the most common HNSCC treatment category (39.64%). Overall, 17.71% of patients had high-risk HPV positive status.

**TABLE 1:** Distribution of baseline demographic characteristics, traditional risk factors, and head and neck squamous cell carcinoma (HNSCC)-related variables by incident cardiovascular disease (CVD) status at 1-year post cancer diagnosis, in patients without prevalent CVD at cancer diagnosis.

	All HNSCC patients—no prevalent CVD (*N* = 1,829)	HNSCC patients—no incident CVD at 1-year (*N* = 1,635)	HNSCC patients -incident CVD at 1-year (*N* = 194)	*p*-value
**Age at HNSCC diagnosis**				
[Median (IQR) years]	61.0 (54.0–68.0)	60.0 (53.0–67.0)	67.0 (61.0–74.0)	**<0**.**001**
**Sex**				
Female	464 (25.37)	415 (25.38)	49 (25.26)	0.97
Male	1,365 (74.63)	1,220 (74.62)	145 (74.74)	
**Race**				
White	1,549 (84.68)	1,378 (84.28)	171 (88.14)	0.25
Black	235 (12.85)	214 (13.09)	21 (10.82)	
Other	45 (2.46)	43 (2.63)	2 (1.03)	
**Marital status**				
Married/with partner	1,059 (57.90)	950 (58.10)	109 (56.19)	0.24
Divorced/separated/widowed	381 (20.83)	332 (20.31)	49 (25.26)	
Single	389 (21.27)	353 (21.59)	36 (18.56)	
**Geographic location (rurality)**				
Urban	1,126 (61.56)	1,013 (61.96)	113 (58.25)	0.32
Rural	703 (38.44)	622 (38.04)	81 (41.75)	
**Alcohol use**				
Current	757 (41.39)	686 (41.96)	71 (36.60)	0.23
Former	141 (7.71)	128 (7.83)	13 (6.70)	
Never	931 (50.90)	821 (50.21)	110 (56.70)	
**Tobacco use**				
Current	602 (32.91)	546 (33.39)	56 (28.87)	0.43
Former	688 (37.62)	609 (37.25)	79 (40.72)	
Never	639 (29.47)	480 (29.36)	59 (30.41)	
**BMI category**				
Non-obese	1,339 (73.21)	1,197 (73.21)	142 (73.20)	0.99
Obese	490 (26.79)	438 (26.79)	52 (26.80)	
**Hypertension at baseline**				
Absent	841 (45.98)	716 (43.79)	125 (64.43)	**<0**.**001**
Present, use medications	966 (52.82)	901 (55.11)	65 (33.51)	
Present, no medication record	22 (1.20)	18 (1.10)	4 (2.06)	
**Dyslipidemia at baseline**				
Absent	1,340 (73.26)	1,215 (74.31)	125 (64.43)	**<0**.**013**
Present, use medications	443 (24.22)	380 (23.24)	63 (32.47)	
Present, no medication record	46 (2.52)	40 (2.45)	6 (3.09)	
**Diabetes at baseline**				
Absent	1,584 (86.60)	1,429 (87.40)	155 (79.90)	**<0**.**012**
Present, use medications	226 (12.36)	191 (11.68)	35 (18.04)	
Present, no medication record	19 (1.04)	15 (0.92)	4 (2.06)	
HNSCC anatomical subsite				**<0**.**014**
Oral cavity	615 (33.62)	536 (32.78)	79 (40.72)	
Oropharynx	602 (32.91)	541 (33.09)	61 (31.44)	
Nasopharynx/nasal cavity	88 (4.81)	80 (4.89)	8 (4.12)	
Hypopharynx	60 (3.28)	56 (3.43)	4 (2.06)	
Larynx	431 (23.56)	397 (24.28)	34 (17.53)	
Salivary	33 (1.80)	25 (1.53)	8 (4.12)	
**Clinical stage at diagnosis**				
Early (stages 0/I/II)	644 (35.21)	572 (34.98)	72 (37.11)	0.54
Advanced (III/IV)	906 (49.54)	817 (49.97)	89 (45.88)	
Other	279 (15.25)	246 (15.05)	33 (17.01)	
**HNSCC treatment category**				
Surgery only	725 (39.64)	628 (38.41)	97 (50.00)	**<0**.**003**
Chemo only	64 (3.50)	63 (3.85)	1 (0.52)	
Radiation only	148 (8.09)	133 (8.13)	15 (7.73)	
Chemoradiation	275 (15.04)	253 (15.47)	22 (11.34)	
Surgery + chemo/rad	347 (18.97)	306 (18.72)	41 (21.13)	
No treatment	270 (14.76)	252 (15.41)	18 (9.28)	
**High-risk HPV status**				
Positive	324 (17.71)	301 (18.41)	23 (11.86)	**<0**.**018**
Negative	243 (13.29)	223 (13.64)	20 (10.31)	
Not tested/reported	1,262 (69.00)	1,111 (67.95)	151 (77.84)	

CVD outcomes = composite of ischemic heart disease, heart failure, and ischemic stroke, whichever occurred first; bold = *p*-value ≤ 0.05.

**FIGURE 2:**
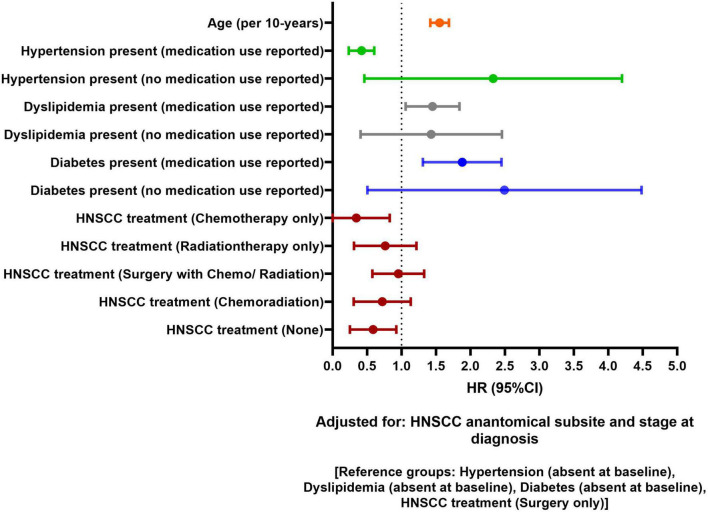
Forest plot showing adjusted association of head and neck squamous cell carcinoma (HNSCC)-related factors and traditional risk factors with 1-year cardiovascular disease (CVD) risk in HNSCC patients.

Figure 2 shows adjusted association of traditional CVD risk factors and HNSCC-related factors with risk of incident CVD at 1-year post HNSCC diagnosis. Per 10-year increase in HNSCC diagnosis age was associated with 57% higher 1-year risk of CVD [HR, 95% CI: 1.57 (1.41–1.68)], after adjusting for hypertension, dyslipidemia, diabetes, HNSCC anatomical subsite, clinical stage at diagnosis, and treatment. Hypertensive patients who used antihypertensive medications at baseline had a lower 1-year risk of incident CVD than patients without hypertension [HR, 95% CI: 0.41 (0.24–0.61)]. Patients with dyslipidemia and diabetes at baseline had higher 1-year CVD risk than patients without the respective CVD risk factors. Compared to patients receiving HNSCC surgery, patients receiving only chemotherapy and no HNSCC treatment had lower 1-year CVD risk in the adjusted model [HR, 95% CI: 0.12 (0.01–0.90) and 0.55 (0.27–0.94), respectively]. No statistically significant association was observed for stage at HNSCC diagnosis and anatomical subsite in the adjusted model. No race or sex-based differences were observed. Unadjusted and adjusted association for 1-year CVD risk is presented in Supplementary Table 2.

**FIGURE 3:**
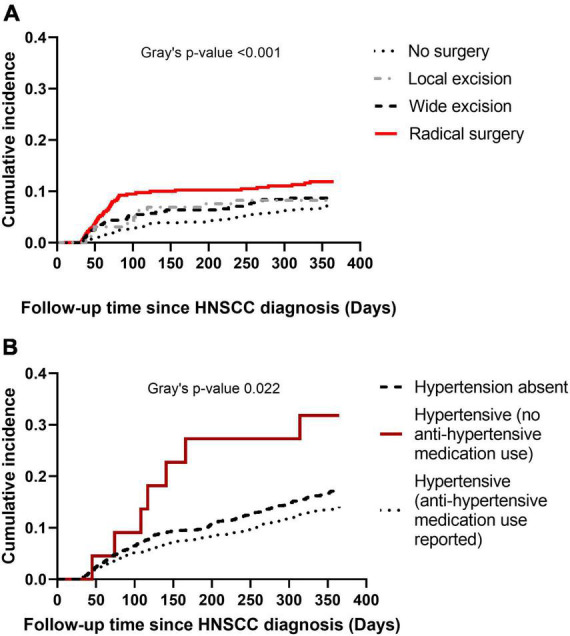
Cumulative incidence plots of cardiovascular disease (CVD) at 1-year post head and neck squamous cell carcinoma (HNSCC) diagnosis. Panel **(A)** shows cumulative incidence of CVD by type of HNSCC surgery. Panel **(B)** shows cumulative incidence of CVD by baseline hypertension.

Cumulative incidence of CVD at 1-year post HNSCC diagnosis varied by HNSCC surgery category (Gray’s *p* < 0.001) (Figure 3A), with a sharp rise in cumulative incidence observed in the first 90 days in the radical surgery group. Cumulative incidence of CVD at 1-year also varied by baseline hypertension (Gray’s *p* 0.022) (Figure 3B). Baseline hypertensive patients who used anti-hypertensive medications had the lowest cumulative incidence of CVD.

**FIGURE 4:**
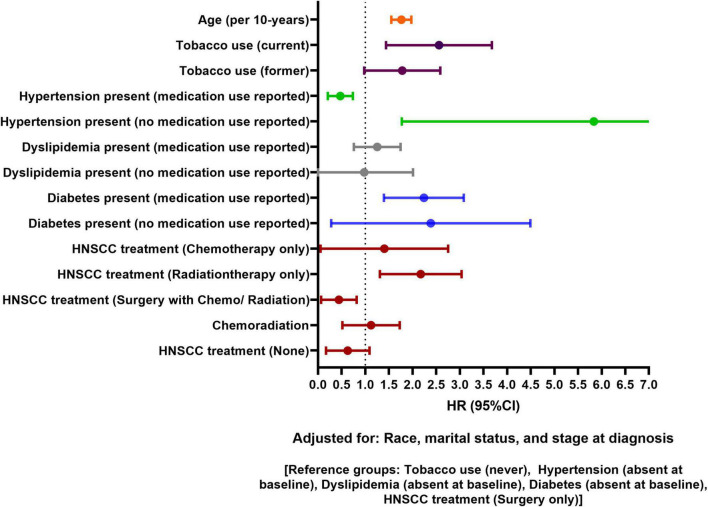
Forest plot showing adjusted association of head and neck squamous cell carcinoma (HNSCC)-related factors and traditional risk factors with 5-year cardiovascular disease (CVD) risk in HNSCC patients.

Data on 5-year follow-up was available for 1,054 HNSCC patients. Figure 4 shows adjusted model for 5-year CVD risk post HNSCC diagnosis. After adjusting for age, race, marital status, baseline tobacco use, stage at diagnosis, hypertension, and diabetes status, HNSCC patients receiving only radiation had 117% higher 5-year risk of CVD than patients receiving only surgery [HR, 95% CI: 2.17 (1.31–3.04)]. Per 10-year increase in age was associated with 79% increase in 5-year risk of CVD [HR, 95% CI: 1.79 (1.54–1.96)]. Tobacco use at baseline (current) was associated with increased 5-year risk of CVD [HR, 95% CI: 2.54 (1.45–3.69)]. Patients who used antihypertensive medications at baseline had lower 5-year CVD risk than patients without hypertension [HR, 95% CI: 0.45 (0.22–0.75)]. Hypertensive patients who did not use antihypertensive medications had higher 5-year CVD risk than patients without hypertension [HR, 95% CI: 5.35 (2.04–10.12)]. Baseline diabetes was associated with increased 5-year CVD risk. Baseline dyslipidemia and stage at HNSCC diagnosis were not associated with 5-year CVD risk. No race or sex-based differences in 5-year risk of CVD were observed. Unadjusted and adjusted association for 5-year CVD risk is presented in Supplementary Table 3.

**FIGURE 5:**
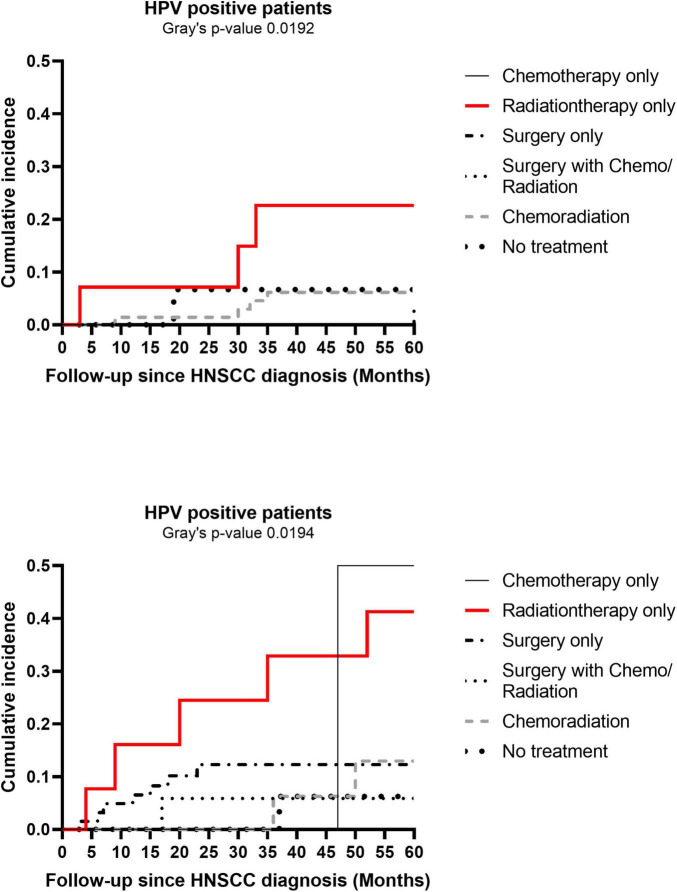
Cumulative incidence plots of cardiovascular disease (CVD) at 5-years post head and neck squamous cell carcinoma (HNSCC) diagnosis by human papillomavirus (HPV) status. Cumulative incidence plots of CVD at 5-years by cancer treatment categories are shown separately for HPV-positive and HPV-negative HNSCC patients.

In sensitivity analysis, per 10-year increase in HNSCC diagnosis age was associated with higher 1-year CVD risk, but not with 5-year risk of CVD, in patients aged < 65 years at baseline (data not shown). Cumulative incidence of CVD events at 5-years post HNSCC diagnosis varied by HNSCC treatment category in both HPV-positive and HPV-negative patients (Gray’s *p* 0.019 in both HPV groups) (Figure 5). While patients receiving only radiation had higher cumulative incidence irrespective of HPV status, overall cumulative incidence of CVD at 5-years was higher in the HPV-negative group than in HPV-positive patients (Gray’s *p* 0.013). Differences in demographics, traditional risk factors, and HNSCC-related factors by HNSCC treatment category are presented in Supplementary Table 4.

## Discussion

This clinical cohort study provides insight into the association of traditional risk factors and cancer-related factors with risk of CVD in patients diagnosed with HNSCC. Antihypertensive medication use at baseline was associated with reduced risk of CVD at 1- and 5-years post HNSCC diagnosis. Older age and diabetes at HNSCC diagnosis increased the risk of CVD at both time-points. Risk of CVD at 1- and 5-years varied by HNSCC treatment category; 1-year CVD risk was high in patients receiving HNSCC surgery, however, patients receiving radiation therapy had higher 5-year CVD risk compared to surgery patients. Compared to HPV-positive patients, HPV-negative patients were older and had a higher 5-year cumulative incidence of CVD.

Peri- and post-operative CVD complications in HNC patients receiving surgery have been reported in literature (16, 17, 24). Older age, extent of surgery, cancer-induced thrombosis, pre-existing comorbidities, previous history of coronary artery disease and heart failure are important predictors of adverse CVD events at 30- and 60-days post HNC surgery (24, 28). In our study, patients receiving HNSCC surgery had high 1-year risk of CVD. Even though immediate post-operative CVD risk was not our outcome of interest, the pattern we observed in cumulative incidence plots suggests association of extent of HNSCC surgery with post-operative CVD risk. Cumulative incidence of CVD was noticeably higher in patients receiving radical surgery during the first 90 days of follow-up; the difference became non-existent at 5-years post HNSCC diagnosis. Increased blood loss and hemodynamic fluctuations following radical surgery may have an influence on acute myocardial infarction. Death as competing risk might partially explain the lack of difference in CVD cumulative incidence at 5-years by surgery type, as patients receiving radical surgery were less likely to be alive by 5-years than other surgery groups (data not shown).

While 1-year CVD risk was lower in patients receiving no HNSCC treatment compared to surgery patients, it is important to note that a higher proportion of HNSCC patients in the “no HNSCC treatment” category had advanced stage at cancer diagnosis and might have died before they developed CVD. Patients receiving radiation therapy had higher 5-year risk of incident CVD than patients receiving surgery; however, no association of radiation therapy with short-term CVD risk was observed. This is not surprising, as previous studies have suggested long latent period between cancer radiation therapy and established atherosclerosis (12, 29). Even though dose-response relationship between HNC radiation and risk of ischemic stroke at 5-years post radiation therapy has been suggested (11), limited data availability restricted us from assessing incident CVD risk based on radiation dose. While a higher risk of myocardial infarction has been suggested in patients receiving chemotherapy (30), we observed no difference in CVD risk based on bolus and weekly administration of platinum-based chemotherapy in patients receiving chemotherapy only. The lack of difference could be due to small sample size in the chemotherapy only group. Five-year risk of CVD was not higher in patients who received chemoradiation compared to surgery patients. This is unexpected as chemoradiation is hypothesized to be associated with higher adverse cardiovascular events. However, in our study population, we observed statistically significant differences in demographics, traditional risk factors, and HNSCC-related factors between the different HNSCC treatment categories. Despite having an advanced TNM stage at HNSCC diagnosis, a higher proportion of patients who received chemoradiation were young, had oropharynx cancer, and had HPV-positive status than patients who received radiation only or surgery only. The combination of these factors could partly explain lower CVD risk in patients who received chemoradiation compared to radiation only patients. This finding also reiterates the lack of association between HNSCC stage at diagnosis and CVD risk.

Association of oncogenic HPV infection with increased risk of cardiovascular events has been suggested in women with vaginal HPV infection (31). Data on HPV and CVD and cerebrovascular event risk in HNC patients, however, are not conclusive. Addison et al. reported four-times higher risk of cerebrovascular events in HPV-positive patients compared to HPV-negative patients receiving radiation therapy (32); they also suggested that difference in cerebrovascular risk by HPV status became evident ∼2 years after radiation therapy and persisted throughout follow-up (32). On the contrary, Eytan et al. reported lower cumulative probability of congestive heart failure, myocardial infarction, and angina in HPV-positive patients at 5-years, with no difference in cumulative probability of stroke by HPV status (33). We also observed a higher 5-year cumulative incidence of CVD in HPV-negative patients than in HPV-positive patients. The cumulative incidence was consistently higher in HPV-negative patients for each HNSCC treatment category than in HPV-positive patients. Like previous studies (2, 34), HPV-positive patients in our study were diagnosed at a younger age and had better survival at 5-years. In sensitivity analysis, higher 5-year CVD risk persisted in HPV-negative patients, after adjusting for age; the association became statistically non-significant when we adjusted for HNSCC treatment. However, we did not adjust for changes in risk factors over time, as well as for overall comorbidity status; it is possible that residual confounding is there. Unlike Addison’s study that assessed association of HPV with stroke and transient ischemic attack (32), our findings were driven by ischemic heart disease as the most common incident CVD outcome; we did not have enough ischemic stroke events to assess the association of HPV status with stroke separately.

Patients who used antihypertensive medications at baseline had significantly lower CVD risk at all time points. Even though association of hypertension and or antihypertensive medication use with incident CVD outcomes has not been reported widely in HNC literature (10), these associations have been reported in other cancer types (22, 35, 36). Strongman et al. reported increased risk of heart failure in non-Hodgkin’s lymphoma patients who did not have hypertension at baseline. In patients with breast cancer, the evidence is somewhat conflicting. While some studies reported beta blockers to be more favorable in reducing chemotherapy induced cardiac events than other antihypertensive medication classes (35–37), others did not report superiority of either while comparing angiotensin converting enzyme inhibitors with beta blockers (38), and angiotensin II receptor blockers with beta blockers (39), Even though our cumulative incidence plot by hypertension status suggests protective effects of antihypertensive medication use, no statistically significant differences were observed in risk of incident CVD by antihypertensive medication types (data not shown). Future studies are needed to assess role of antihypertensive medications in reducing CVD risk in large, diverse HNSCC populations. Unlike antihypertensive medications, we did not observe any cardio-protective effects of lipid lowering medications or antidiabetic medications on incident CVD risk. Patients with diabetes at baseline had consistently higher CVD risk than patients without diabetes.

In all our models, age was a strong predictor of incident CVD. When stratified by age group, older age was associated with 1-year risk of incident CVD, but not with 5-year CVD risk in patients aged < 65 years. Age is not a modifiable risk factor, but the fact that 5-year risk of incident CVD did not increase with age in patients < 65 years has important clinical implications. Proactive screening and monitoring of modifiable risk factors in patients aged < 65 years can reduce the burden of CVD. Role of obesity in HNC patients is conflicting. While some studies report inverse association of BMI with HNC risk and survival (40, 41); others have identified obesity to be a risk factor for HNC (42). We did not observe any association between obesity and incident CVD in our study population, however, it would be too early to conclude lack of association based on BMI as the only indicator of adiposity. Patients who continue smoking post HNC therapy have a poorer prognosis (43). While current tobacco users had higher 5-year risk of CVD compared to never users, no association was observed between tobacco use and 1-year CVD risk. It is possible that tobacco use was under-reported in our study. It is also possible that altered tobacco habit following HNSCC diagnosis might have impacted CVD risk to some extent (44). However, more studies are needed to determine if the same is true for tobacco use in other HNSCC populations.

Our study had some limitations. Like any other cancer registries and EHR databases, information of HNSCC stage at diagnosis and treatment was incomplete for few patients. We did not have data available on radiation therapy dose, fractionation, or techniques; but these are limitations of using real-world clinical data that we could not address in our study. Under-reporting of CVD risk factors and CVD outcomes in the EHR is also possible, as not all cancer patients come to UAB for their primary care. We did not have information on amount and frequency of tobacco and alcohol use. However, differential bias is less likely as we included EHR data on consecutive HNSCC patients from all UAB clinics. Since ischemic heart disease was the most common incident CVD event in our study population; we did not have enough statistical power to assess risk of heart failure and ischemic stroke separately. Also, ischemic heart disease represents a broad spectrum of presentations ranging from asymptomatic stable coronary disease to acute myocardial infarction. Limited sample size restricted us from further delineating acute events.

Despite the noted limitations, our study provides insight in the complicated association of cancer-related factors and traditional risk factors with incident CVD risk in HNSCC patients. Confirmation of clinical data using medical charts improved validity of our findings. Our results highlight plausible association of antihypertensive medication use with risk of incident CVD in HNSCC patients and may help in identifying HNSCC patients who are at high risk of incident CVD during follow-up. Future research should focus on prophylactic use of antihypertensive, lipid-lowering, and antidiabetic medications, and role of HPV in CVD risk in HNSCC patients.

## Data availability statement

The data analyzed in this study is subject to the following licenses/restrictions: Data are not publicly available. The datasets analyzed during the current study are available from the corresponding author on reasonable request. Requests to access these datasets should be directed to AM, amrita.x.mukherjee@kp.org..

## Ethics statement

The studies involving human participants were reviewed and approved by the University of Alabama at Birmingham (UAB), Institutional Review Board (IRB), O’Neal Comprehensive Cancer Center Tumor Registry. Written informed consent for participation was not required for this study in accordance with the national legislation and the institutional requirements.

## Author contributions

AM and SS contributed to the conception of research hypothesis, study design, and data acquisition. AM contributed to the data analysis, interpretation, and manuscript writing. SS, HW, and RG provided the feedback on analysis and interpretation of results. AM and AC contributed to the adjudication of clinical risk factors. CL, AC, LN, and CEL critically reviewed the manuscript and provided the clinical expertise in interpretation of results. All authors gave their final approval and are responsible for the content of the manuscript.
