# Supplementary material for: Traditional risk factors and cancer-related factors associated with cardiovascular disease risk in head and neck cancer patients
Source: Front Cardiovasc Med. 2023 Jan 12;9:1024846. doi: 10.3389/fcvm.2022.1024846 (PMC9877509; doi:10.3389/fcvm.2022.1024846)
Supplement: Supplementary file 1 [file Table_1.docx]

| **Supplementary Table S1: ICD-9/ 10 codes and medications used to identify CVD clinical risk factors (hypertension, dyslipidemia, and diabetes) in HNSCC patients** | | |
| --- | --- | --- |
| **CVD Clinical risk factors** | **ICD-9/ 10 codes** | **Medications** |
| **Hypertension** | 401., I10. | Diuretics other than Loop, Beta blockers, Angiotensin-converting enzyme inhibitors, Angiotensin receptor blockers, Calcium channel blockers, Combined alpha and beta blockers, Central agonists, vasodilators |
| **Dyslipidemia** | 272.0 – 272.5, E78.0 – E78.6 | HMG CoA reductase inhibitors, Bile acid sequestrants, Fibrates, Niacin, PCSK9 inhibitors, Ezetimibe |
| **Diabetes** | 250., E10., E11. | Biguamides, Dipeptidyl peptidase 4, GLP-1 receptor agonists, Alpha glucosidase inhibitors, Meglitinides, Sulphonylureas, SGLT-2 inhibitors, Thiazolidinediones, Insulin |

| **Supplementary Table S2: Unadjusted and adjusted models for risk of incident CVD at one-year post HNSCC diagnosis in patients without prevalent CVD at cancer diagnosis** | | |
| --- | --- | --- |
| **N=1829** | **Unadjusted model**  **HR (95%CI)** | **Adjusted model***  **HR (95% CI)** |
| **Age at HNSCC diagnosis**  **(per 10-years**) | **1.66 (1.52-1.76)** | **1.57 (1.41-1.68)** |
| **Sex**  Male  Female | Ref  0.99 (0.68-1.32) | NA |
| **Race**  White  Black  Other | Ref  0.81 (0.47-1.23)  0.39 (0.07-1.48) | NA |
| **Marital Status**  Single  Married/ with partner  Divorced/ separated/ widowed | Ref  1.08 (0.71-1.49)  1.40 (0.90-1.96) | NA |
| **Geographic location (rurality)**  Urban  Rural | Ref  1.17 (0.86-1.47) | NA |
| **Alcohol Use**  Never  Current  Former | Ref  0.78 (0.53-1.04)  0.74 (0.37-1.27) | NA |
| **Tobacco Use**  Never  Current  Former | Ref  0.87 (0.56-1.22)  1.05 (0.72-1.40) | NA |
| **BMI Category**  Non-obese  Obese | Ref  0.98 (0.68-1.29) | NA |
| **Hypertension at baseline**  Absent  Present (with medication use)  Present (without medication use) | Ref  **0.44 (0.27-0.64)**  1.27 (0.42-2.94) | Ref  **0.41 (0.24-0.61)**  1.95 (0.68-4.36) |
| **Dyslipidemia at baseline**  Absent  Present (with medication use)  Present (without medication use) | Ref  **1.57 (1.17-1.94)**  1.39 (0.57-2.72) | Ref  **1.45 (1.06-1.84)**  1.26 (0.50-2.53) |
| **Diabetes at baseline**  Absent  Present (with medication use)  Present (without medication use) | Ref  **1.65 (1.16-2.16)**  2.35 (0.85-5.03) | Ref  **1.88 (1.31-2.45)**  2.10 (0.73-4.65) |
| **HNSCC treatment category**  Surgery only  Chemotherapy only  Radiation therapy only  Chemoradiation  Surgery with chemo or radiation  No treatment | Ref  **0.11 (0.01-0.81)**  0.71 (0.36-1.19)  **0.65 (0.40-0.96)**  0.88 (0.52-1.32)  **0.52 (0.26-0.88)** | Ref  **0.12 (0.01-0.90)**  0.71 (0.34-1.24)  0.66 (0.34-1.16)  0.93 (0.59-1.34)  **0.55 (0.27-0.94)** |
| **HNSCC anatomic subsite**  Oral cavity  Oropharynx  Nasopharynx/ nasal cavity  Hypopharynx  Larynx  Salivary | Ref  0.76 (0.50-1.06)  0.71 (0.29-1.41)  0.49 (0.14-1.30)  **0.58 (0.34-0.89)**  **2.15 (1.04-3.69)** | Ref  1.17 (0.77-1.60)  0.79 (0.33-1.55)  0.74 (0.22-1.91)  0.76 (0.45-1.18)  1.70 (0.78-3.07) |
| **Clinical Stage at diagnosis**  Early (Stages 0/ I/ II)  Advanced (III/ IV)  Other | Ref  0.90 (0.62-1.20)  1.07 (0.67-1.52) | Ref  1.15 (0.79-1.52)  0.96 (0.59-1.40) |
| Notes:  * Adjusted for clinical stage at diagnosis, and for variables with p-value ≤0.10 in unadjusted models  NA- not included in model  Bold- p-value ≤0.05 | | |

| **Supplementary Table S3: Unadjusted and adjusted models for risk of incident CVD at five-years post HNSCC diagnosis in patients without prevalent CVD at cancer diagnosis** | | |
| --- | --- | --- |
| **N=1054** | **Unadjusted model**  **HR (95%CI)** | **Adjusted model***  **HR (95% CI)** |
| **Age at HNSCC diagnosis**  **(per 10-years**) | **1.69 (1.48-1.84)** | **1.79 (1.54-1.96)** |
| **Sex**  Male  Female | Ref  0.99 (0.58-1.44) | NA |
| **Race**  White  Black  Other | Ref  **1.76 (1.09-2.44)**  0.70 (0.12-2.41) | Ref  1.34 (0.74-2.01)  1.062 (0.20-3.48) |
| **Marital Status**  Single  Married/ with partner  Divorced/ separated/ widowed | Ref  0.65 (0.33-1.04)  1.22 (0.67-1.84) | Ref  0.75 (0.39-1.21)  1.11 (0.58-1.75) |
| **Geographic location (rurality)**  Urban  Rural | Ref  1.11 (0.71-1.51) | NA |
| **Alcohol Use**  Never  Current  Former | Ref  0.84 (0.50-1.22)  0.88 (0.34-1.73) | NA |
| **Tobacco use status (baseline)**  Never  Current  Former | Ref  **1.98 (1.13-2.89)**  **1.87 (1.08-2.70)** | Ref  **2.54 (1.45-3.69)**  1.76 (0.99-2.60) |
| **BMI Category**  Non-obese  Obese | Ref  0.70 (0.37-1.10) | NA |
| **Hypertension at baseline**  Absent  Present (with medication use)  Present (without medication use) | Ref  **0.49 (0.26-0.79)**  2.54 (0.90-5.13) | Ref  **0.45 (0.22-0.75)**  **5.35 (2.04-10.12)** |
| **Dyslipidemia at baseline**  Absent  Present (with medication use)  Present (without medication use) | Ref  1.50 (0.99-1.98)  1.10 (0.28-2.85) | Ref  1.25 (0.76-1.75)  0.67 (0.13-2.13) |
| **Diabetes at baseline**  Absent  Present (with medication use)  Present (without medication use) | Ref  **1.93 (1.21-2.62)**  **3.52 (1.32-6.77)** | Ref  **2.25 (1.39-3.08)**  1.94 (0.54-4.68) |
| **HNSCC treatment category**  Surgery only  Chemotherapy only  Radiation therapy only  Chemoradiation  Surgery with chemo or radiation  No treatment | Ref  1.22 (0.31-3.16)  **2.47 (1.55-3.35)**  1.07 (0.57-1.65)  **0.37 (0.12-0.84)**  0.69 (0.28-1.31) | Ref  1.06 (0.26-2.89)  **2.17 (1.31-3.04)**  1.08 (0.54-1.75)  **0.37 (0.11-0.85)**  0.56 (0.21-1.12) |
| **HNSCC anatomic subsite**  Oral cavity  Oropharynx  Nasopharynx/ nasal cavity  Hypopharynx  Larynx  Salivary | Ref  0.67 (0.34-1.09)  1.12 (0.44-2.17)  0.92 (0.26-2.22)  0.93 (0.51-1.43)  1.12 (0.21-3.63) | NA |
| **Clinical Stage at diagnosis**  Early (Stages 0/ I/ II)  Advanced (III/ IV)  Other | Ref  1.14 (0.70-1.60)  1.55 (0.84-2.35) | Ref  1.58 (0.96-2.21)  1.71 (0.92-2.60) |
| Notes:  * Adjusted for clinical stage at diagnosis, and for variables with p-value ≤0.10 in unadjusted models  NA- not included in model  Bold- p-value ≤0.05 | | |

| **Supplementary Table S4: Distribution of baseline demographic characteristics, traditional risk factors and HNSCC-related variables by HNSCC treatment category** | | | | | | | |
| --- | --- | --- | --- | --- | --- | --- | --- |
|  | **Surgery only**  **(%)** | **Chemotherapy only**  **(%)** | **Radiation only**  **(%)** | **Chemo**  **Radiation**  **(%)** | **Surgery with chemo/ rad**  **(%)** | **No treatment**  **(%)** | **p-value** |
| **Age at HNSCC diagnosis**  [Median (IQR) years] | 59.0  (51.0-66.0) | 60.0  (53.5-65.0) | 61.0  (55.0-70.0) | 57.0  (52.0-64.0) | 59.0  (51.0-66.0) | 62.0  (55.0-68.0) | **<0.001** |
| **Sex**  Female  Male | 31.86  68.14 | 14.06  85.94 | 23.65  76.35 | 16.00  84.00 | 24.21  75.79 | 22.59  77.41 | **<0.001** |
| **Race**  White  Black  Other | 88.14  9.66  2.21 | 64.06  32.81  3.13 | 79.05  18.24  2.70 | 85.82  12.00  2.18 | 87.03  11.53  1.44 | 79.26  16.30  4.44 | **<0.001** |
| **Tobacco Use**  Current  Former  Never | 31.45  35.72  32.83 | 40.63  42.19  17.19 | 34.46  39.19  26.35 | 33.82  41.09  25.09 | 28.53  39.77  31.70 | 38.89  34.44  26.67 | **0.034** |
| **BMI Category**  Non-obese  Obese | 71.86  28.14 | 79.69  20.31 | 77.70  22.30 | 70.91  29.09 | 73.78  26.22 | 74.44  25.56 | 0.470 |
| **Hypertension at baseline**  Absent  Present, use medications  Present, no medication record | 49.79  49.79  0.41 | 45.31  51.56  3.13 | 45.95  52.03  2.03 | 44.73  53.09  2.18 | 43.23  56.20  0.58 | 40.74  57.04  2.22 | **0.032** |
| **Dyslipidemia at baseline**  Absent  Present, use medications  Present, no medication record | 74.62  22.76  2.62 | 78.13  18.75  3.13 | 62.16  35.81  2.03 | 73.82  23.27  2.91 | 76.66  21.61  1.73 | 69.63  27.41  2.96 | 0.069 |
| **Diabetes at baseline**  Absent  Present, use medications  Present, no medication record | 88.69  10.48  0.83 | 92.19  7.81  0.00 | 84.46  12.16  3.38 | 85.82  12.36  1.82 | 86.46  13.26  0.29 | 81.85  17.41  0.74 | **0.011** |
| **HNSCC anatomical subsite**  Oral cavity  Oropharynx  Nasopharynx/ nasal cavity  Hypopharynx  Larynx  Salivary | 54.76  18.90  4.41  0.69  19.03  2.21 | 9.38  51.56  10.94  6.25  21.88  0.00 | 5.41  36.49  2.03  9.46  45.95  0.68 | 5.82  53.82  7.64  5.45  27.27  0.00 | 38.90  35.73  4.03  1.73  15.56  4.03 | 19.63  39.26  4.07  5.93  30.37  0.74 | **<0.001** |
| **Clinical Stage at diagnosis**  Early (Stages 0/ I/ II)  Advanced (III/ IV)  Other | 51.72  28.97  19.31 | 21.88  62.50  15.63 | 47.30  43.92  8.78 | 7.64  83.27  9.09 | 18.73  67.44  13.83 | 36.67  47.41  15.93 | **<0.001** |
| **High-risk HPV status**  Positive  Negative/Not reported | 9.10  90.90 | 20.31  79.69 | 16.22  83.78 | 36.00  64.00 | 24.78  75.22 | 13.33  86.67 | **<0.018** |
| Notes: p-values reported are from Chi-square tests. | | | | | | | |
